# Prevalence of *Cryptosporidium* in La Trinidad, Benguet, Philippines: a One Health approach

**DOI:** 10.3389/fpara.2025.1557608

**Published:** 2025-03-14

**Authors:** Jannette Depay Awisan, Pilarita Tongol Rivera, Jose Ma. Moncada Angeles

**Affiliations:** ^1^ Department of Medical Laboratory Science, School of Nursing, Allied Health, and Biological Sciences, Saint Louis University, Baguio, Philippines; ^2^ Department of Parasitology, University of the Philippines-Manila, Manila, Philippines

**Keywords:** *Cryptosporidium*, zoonotic diseases, La Trinidad, Benguet, public health, parasitology, Philippines

## Abstract

**Introduction:**

*Cryptosporidium* species are zoonotic protozoa responsible for cryptosporidiosis, a serious public health concern for humans and animals. These protozoa are recognized for their capacity to infect various hosts, resulting in outbreaks that can cause significant health and economic consequences. The One Health approach considers human, animal, and environmental health interconnectedness and is vital in understanding and controlling the spread of such zoonotic diseases. This study adopts this approach to evaluate the prevalence of *Cryptosporidium* in humans, companion animals, livestock, and environmental water sources in La Trinidad, Benguet.

**Methods:**

A cross-sectional descriptive study was conducted from September 2020 to January 2022, adhering to research ethical standards approved by the Institutional Review Board (IRB) and following COVID-19 safety protocols such as social distancing, use of PPE, and regular sanitation of equipment and facilities. Stratified random sampling resulted in 314 participating households, which provided fecal samples from humans (up to two members), companion animals, and livestock. Samples were analyzed using microscopy (Sugar Flotation Technique, Formalin Ether Concentration Technique, and Kinyoun staining) and molecular methods, with genomic DNA extracted and nested PCR targeting the 18S rRNA gene. Water samples from 19 community sites underwent filtration and nested PCR analysis.

**Results:**

From the 493 human, 363 animal, and 19 water samples analyzed, microscopic analysis revealed that 151 samples tested positive for *Cryptosporidium* oocysts, and molecular confirmation identified 135 (15.77%) as *Cryptosporidium parvum*. Livestock exhibited the highest prevalence (37.27%), followed by companion animals (18.58%) and humans (9.33%), indicating significant zoonotic transmission risks and highlighting the need for improved biosecurity measures. All water samples were negative.

**Discussion:**

The high burden of *Cryptosporidium* in livestock presents significant risks for zoonotic transmission and reflects major shortcomings in biosecurity and sanitation. In contrast, the low human prevalence of COVID-19 suggests that enhancing hygiene practices combined with social restraint may help control infectious events. Further research is required to confirm this relationship. These results highlight the need for targeted public health interventions to reduce transmission risks.

## 1 Introduction

*Cryptosporidium* species are zoonotic protozoa that cause the disease cryptosporidiosis, which presents an important health concern for both humans and animals. These parasites are mainly transmitted through water, food, or direct fecal-oral contact with infected populations, leading to significant public health concerns, particularly in areas with frequent human-animal interaction. *Cryptosporidium* also has high prevalence for human and animal infections, ranging from 10.5% to 71.45% and 2.61% to 73.33%, respectively. These prevalences are notably higher in children and immunocompromised individuals, particularly susceptible to severe cryptosporidiosis (Pumipuntu and Piratae, 2018; Labana, 2018). In the Philippines, *Cryptosporidium* prevalence among humans has been reported to range from 1.9% to 36%, with community-based studies showing higher prevalence than hospital-based studies (Labana, 2018; Natividad et al., 2008; Rivera and Anthony, 2009).

Due to diagnostic limitations, prevalences of *Cryptosporidium* infections are often underestimated, especially among immunocompromised populations. Conventional diagnostic methods usually miss *Cryptosporidium* oocysts because of their small size. This leads to underreporting, especially in areas with a high prevalence of diseases such as HIV/AIDS and cancer (Pane and Putignani, 2022; O’Leary et al., 2021). In such settings, household-based prevalence studies can provide more accurate insights into infection, helping to inform targeted health interventions and guide public Health and veterinary policies to enhance diagnostic capabilities and reduce exposure through improved water sanitation and animal waste management strategies (Newman, 1994).

In the Philippines, various animal reservoirs, including livestock, companion animals, and wildlife, facilitate the zoonotic transmission of *Cryptosporidium*. These animals contribute to environmental contamination, which can occur through food, and water increasing risks in communities with frequent human-animal interactions (Labana, 2018). Extensive documentation exists for waterborne outbreaks of *Cryptosporidium* in developed countries, but the Philippines reports these outbreaks less frequently, mainly due to inadequate surveillance infrastructure and underreporting (Masangkay et al., 2020). In contrast, neighboring Southeast Asian countries such as Malaysia have advanced their detection efforts through molecular epidemiological studies. However, research on *Cryptosporidium* in the Philippines remains sparse (Labana, 2018).

Despite global advances in *Cryptosporidium* research, researchers have notably neglected studies in the Philippines, particularly in the Cordillera Administrative Region (CAR), where there are minimal *Cryptosporidium* prevalence studies. The town of La Trinidad, Benguet, which is experiencing rapid urbanization, presents a critical gap in research. Many households in this area depend on untreated water sources, which raises the risk of waterborne diseases. Although water from refilling stations undergoes basic bacteriological testing, pathogens such as *Cryptosporidium* and *Giardia* often go undetected, creating a potential reservoir for infection (Labana, 2018).

Using a One Health approach, the study aims to determine the prevalence of Cryptosporidium among humans, animals, and water sources in La Trinidad, Benguet. This framework integrates human, animal, and environmental health perspectives, emphasizing the interconnectedness of these sectors in understanding zoonotic diseases like *Cryptosporidium*, where human, animal, and environmental factors play a central role in transmission dynamics (Innes et al., 2020). In La Trinidad, where individuals closely interact with livestock and companion animals, the potential for *Cryptosporidium* transmission is high, as these interactions expose humans to oocysts, promoting zoonotic transmission. Water sources in La Trinidad vary, with 97.4% of households accessing Level III water from the La Trinidad Water District (LTWD) and community cooperatives. However, 1,259 households still rely on Level I sources, including shallow and deep wells, and 54.98% use bottled or purified water for drinking. These diverse water sources and household practices provide essential context for public health studies examining waterborne diseases like *Cryptosporidium* infections. The barangay characteristics and water usage patterns highlight areas of potential risk for infection (Municipality of La Trinidad – Benguet, Philippines, n.d.). Investigating these dynamics at the household level offers valuable insights into direct sources of infection and enables targeted interventions to reduce the risk of disease spread.

Integrating clinical, veterinary, and environmental data, the One Health framework provides a comprehensive view of disease transmission and control (Innes et al., 2020). This study will enhance disease surveillance and prevention strategies, identifying key factors contributing to Cryptosporidium transmission in La Trinidad. Moreover, the findings will contribute to a deeper understanding of cryptosporidiosis epidemiology in the Philippines, supporting local public health efforts to improve water safety, sanitation, and surveillance practices, ultimately helping eradicate Cryptosporidium’s spread.

## 2 Materials and methods

This cross-sectional descriptive study evaluated the prevalence of *Cryptosporidium* in human, animal, and environmental samples in La Trinidad, Benguet, from September 2020 to June 2022. The research team secured ethical clearance from the University of the Philippines Manila Research Ethics Board (UPMREB 2020-255-01) and adhered to all COVID-19 safety protocols.

### 2.1 Study area and sampling

**Figure 1:**
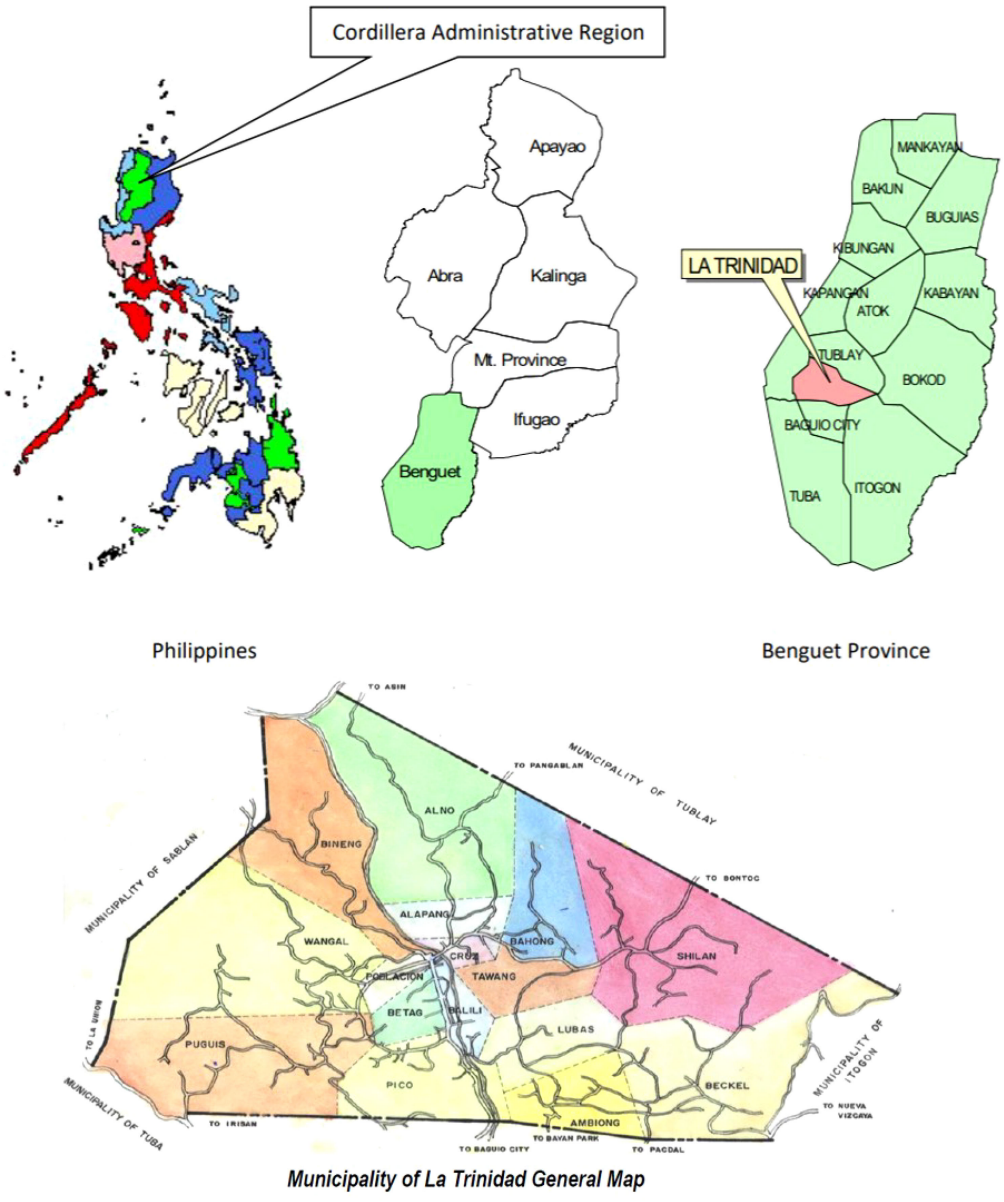
Map of La Trinidad, Benguet, Philippines, adapted from the Municipality of La Trinidad (https://latrinidad.gov.ph).

The study was conducted in La Trinidad, Benguet (**Figure 1**), a municipality with both urban and rural areas. Households were selected using a stratified random sampling method based on eligibility criteria, including residence in the household for at least one year. Each household provided fecal/stool samples from up to two members, one companion animal, and one livestock. For human samples, priority was given based on the following hierarchy: (a) a child aged five years or below (youngest, but not less than six months), (b) a child aged 18 years or below, (c) a senior aged 60 years or above, (d) the primary food preparer, and (e) the primary financial provider. Individuals who had taken anti-diarrheal drugs, mineral oil-based laxatives, or antimicrobial agents within two weeks were excluded. However, the exclusion of an individual did not disqualify the entire household. For companion animals, if multiple were present, one dog was randomly selected; in the absence of a dog, another animal was chosen randomly. A similar random selection process was applied to livestock, prioritizing cattle followed by other species. Participants were informed about the study details, data collection procedures, and their right to withdraw. Written informed consent was obtained, and participants were provided detailed written information about the study.

The sample selection and recruitment for the study were conducted across 16 barangays in La Trinidad, Benguet, using proportionate sampling based on the total number of households in each barangay as of 2015. Researchers calculated a minimum sample size of 352 households and proportionately allocated it across the barangays. Researchers invited 418 households to participate to account for potential non-consent. Among these, 355 households provided consent, but researchers ultimately included only 314. The primary reason for withdrawal was the failure to submit stool samples despite three follow-up visits. In total, researchers collected 493 human stool samples from 288 households (91.72%), 253 companion animal fecal samples from 193 households (61.46%), and 110 livestock fecal samples from 98 households (31.21%). Researchers also collected nineteen water samples for testing.

### 2.2 Fecal sample collection and processing

The researchers collected fecal samples from two human members per household, one companion animal (if present), and one livestock animal (if applicable). Each household received a collection kit containing labeled containers, illustrated instructions, and a cold pack for sample preservation. The researchers instructed the household heads on proper collection, labeling, and storage at 4°C. The household heads retrieved samples within 24 hours, excluding improperly labeled or contaminated samples. All samples were transported on ice to the laboratory and processed within one week.

Fecal samples were analyzed using the Sugar Flotation Technique and the Formalin Ether Concentration Technique (FECT) to isolate *Cryptosporidium* oocysts (Weber et al., 1992; WHO, 1994; Garcia et al., 1983). For the Sugar Flotation Technique, researchers mixed approximately 1.5 grams of stool with water, filtered it, and centrifuged at 500 x g for 10 minutes with a sucrose solution. Researchers collected the supernatant for further examination. In the FECT method, researchers mixed 1.5 grams of stool samples with 10% formalin, filtered the mixture, and centrifuged at 1500 rpm for 3 minutes. Concentrated samples from both techniques were smeared onto glass slides, fixed with absolute methanol, and stained using the Kinyoun staining method for microscopic evaluation of *Cryptosporidium* oocysts.

### 2.3 Water sample collection and processing

Researchers collected water samples from 19 community sources, including protected wells and springs. Five-gallon samples were obtained in sterilized bottles from primary reservoirs and transported to the laboratory for analysis. Upon arrival, samples were stored at 4°C and analyzed within 24 hours.

For processing, samples were maintained at 20 to 25°C and allowed to settle undisturbed for 24 hours. Researchers siphoned off half of each sample volume to minimize sediment disturbance while thoroughly mixing the remaining liter. Sediments were filtered using a Whatman glass microfiber filter (1.2 µm pore size) with a porcelain funnel and rotor. The sediments were eluted from filters into 15-mL centrifuge tubes and centrifuged at 1500 g for 15 minutes (Masangkay, 2020). The resulting pellet was resuspended in sterile phosphate-buffered saline (PBS) and washed twice with 200 µL of sterile PBS. Researchers transferred the sediment to Eppendorf tubes and stored it at -20°C. Water sample processing was conducted from September 2021 to February 2022.

### 2.4 Molecular testing

The researchers conducted DNA extraction on all microscopically positive stool/fecal samples and all 19 water samples for molecular analysis. Researchers isolated genomic DNA using E.Z.N.A. kits, following the manufacturer’s protocols. Researchers detected *Cryptosporidium* species using a nested PCR assay targeting the 18S rRNA gene. Two primers are Cry18S-S2 (5’-GGTGACTCATAATAACTTTACGG-3’) and Cry18S-As (5’-ACGCTATTGGAGCTGGAATTAC-3’) for the first amplification round and Cry18S-S1 (5’-TAAACGGTAGGGTATTGGCCT-3’) and Cry18S-As1 (5’-CAGACTTGCCCTCCAATTGATA-3’) for the second round. Each 25 µL PCR reaction contained 0.5 µM of both forward and reverse primers, 1× Taq Master Mix (Vivantis), and approximately two ng/µL of DNA template, with nuclease-free water added to reach the final reaction volume. For nested PCR, the second amplification used two ng/µL of the first-round amplicon as the template.

The PCR cycling conditions included an initial denaturation at 95°C for 5 minutes, followed by 35 cycles of denaturation at 94°C for 30 seconds, annealing at 65°C for 1 minute, and extension at 72°C for 10 minutes. In the second round, researchers reduced the annealing temperature to 61°C. Researchers used *C. hominis* (UKH135), a reference isolate from stool samples of confirmed human cases, and *C. parvum* (UKP207), obtained from confirmed bovine cases, as positive controls. The *Cryptosporidium* Reference Unit, Public Health Wales, Singleton Hospital, Swansea, UK, provided both control isolates, and we maintained them under sterile conditions for PCR validation. A negative control, molecular biology-grade water, was also included. Human, companion animal, and companion animal samples were analyzed using the same positive control. PCR products were analyzed by gel electrophoresis on 1% agarose gels, visualized under UV light using Cleaver Scientific 1 kb as a reference marker. Positive samples underwent DNA purification and sequencing at Macrogen, Inc., South Korea.

### 2.5 Species identification

Species identification was performed by sequencing the PCR products. The obtained sequences were aligned and compared to reference sequences using the Basic Local Alignment Search Tool (BLAST) against the NCBI GenBank database to confirm the presence and species of *Cryptosporidium*. Results were cross-verified with previously published sequences for validation. Researchers submitted the sequences to NCBI to assign the accession numbers.

## 3 Result

**Figure 2:**
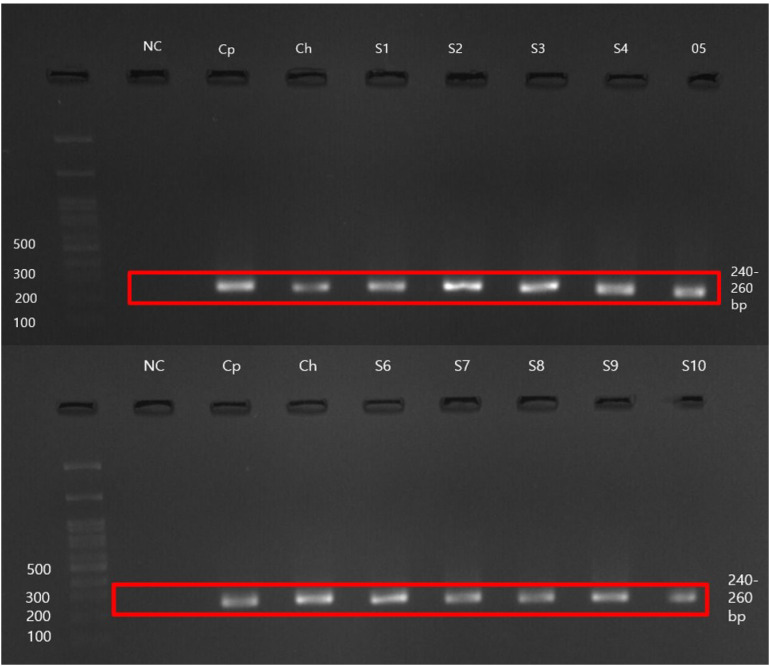
Representative gel electrophoresis image was captured using Viber Gel Documentation Imaging. NC, Negative control; Cp, *Cryptosporidium parvum* positive control (UKP207); Ch, *Cryptosporidium hominis* positive control (UKH135); S1–S10, Analyzed samples.

Researchers identified 151 samples as positive for *Cryptosporidium* oocysts among the microscopically examined samples. Researchers conducted molecular testing on the microscopy-positive samples and 19 filtered water samples. Gel electrophoresis (**Figure 2**) revealed that seven samples did not produce detectable bands. Ten samples failed to generate consensus sequences after DNA sequencing. Through BLAST analysis, we confirmed that 135 (15.77%) samples tested positive for *Cryptosporidium* as *C. parvum*. The nucleotide sequences of the nested PCR products showed a high sequence similarity (97.19% to 100.00%) with reference sequences.

**Table 1** presents a detailed breakdown of *Cryptosporidium* prevalence across various La Trinidad and Benguet categories. The percentages per group show the percentage of samples positive for *C. parvum*, which gives valuable information about the distribution of the protozoan among humans, companion animals, livestock, and water sources. Moreover, less than 1 in 10 (9.33%) of the members of the household tested positive, while 18.58% (47 of 253) of companion animals and 37.27% (41 of 110) of livestock tested positive. For example, the most prevalent source of infection with *Cryptosporidium* among livestock from La Trinidad, Benguet, was more than 2-fold higher than that of companion animals and human members of households containing the different animals.

**Table 1:** Distribution of *Cryptosporidium parvum* in La Trinidad, Benguet.

	N	Microscopy (+)		Molecular Test (+)	
		n	%	n	%
Human samples	493	52	10.55	46	9.33
Companion animal samples	253	57	22.53	47	18.58
Dogs	236	52	22.03	43	18.22
Cats	14	3	21.43	2	14.29
Dove	4	2	50.00	2	50.00
Livestock	110	42	38.18	41	37.27
Chicken	80	30	37.50	30	37.50
Rabbit	12	5	41.67	4	33.33
Pig	10	4	40.00	4	40.00
Cow	4	3	75.00	3	75.00
Duck	3	0	0.00	0	0.00
Horse	1	0	0.00	0	0.00
Water samples	19			0	0.00

Microscopy generally reported higher detection rates than molecular testing across most sample categories. Specifically, microscopy detected 10.55% for human samples, while molecular testing detected 9.33%, showing a 1.22% higher detection rate with microscopy. In companion animals, microscopy identified 22.53%, compared to 18.58% with molecular testing, resulting in a 3.95% higher detection rate for microscopy. For livestock, microscopy detected 38.18%, while molecular testing found 37.27%, yielding a 0.91% higher detection rate with microscopy.

Using the chi-square test, the prevalence of positive samples across different sources shows a statistically significant association between sample sources (χ2 = 60.02,p<0.001, suggesting that the frequency of positive and negative results varied significantly across groups. *Post hoc* analysis using Tukey’s Honest Significant Difference (HSD) test further identified specific pairwise differences. The proportion of positive human samples did not significantly differ from those in companion animals (p > 0.05). However, significant differences were observed between humans and livestock (p < 0.05) and humans and water samples (p < 0.05), indicating that livestock and water sources had distinct patterns of positivity relative to human samples. Similarly, companion animals differed significantly from livestock and water samples (p < 0.05), reinforcing that transmission dynamics or environmental contamination patterns may vary across these categories.

The study investigated the association between companion animals, livestock, and *Cryptosporidium* positivity among household members (**Table 2**). Findings indicate that the overall presence of companion animals did not significantly increase the risk of *Cryptosporidium* positivity (p = 1.00, OR = 1.02). However, when companion animals tested positive for *Cryptosporidium*, household members were significantly more likely to be infected (p = 0.04, OR = 2.55).

**Table 2:** Association of the presence of animals and animals positive with Cryptosporidium *to household member* Cryptosporidium positivity.

	Household member/s		Total	p-value	odds ratio
	*Cryptosporidium* (+)	*Cryptosporidium* (-)			
Companion animals in the household					
without companion animals	10	61	71	1.00	1.02
with companion animals	31	186	217		
Companion animal (+) with *Cryptosporidium*	11	33	44	0.04	2.55
Companion animal (-) with *Cryptosporidium*	20	153	173		
Livestock in household					
without livestock	26	144	170	0.38	1.24
with livestock	15	103	118		
livestock (+) with *Cryptosporidium*	3	36	39	0.41	0.47
livestock (-) with *Cryptosporidium*	12	67	79		

The presence of livestock in the household did not show a statistically significant association with *Cryptosporidium* positivity among household members (p = 0.38, OR = 1.24). Individuals in households with *Cryptosporidium*-positive livestock exhibited a lower, albeit non-significant, risk of infection (p = 0.41, OR = 0.47).

The primary source of household water in the study area is the La Trinidad Water District, which employs regular and closely monitored treatment protocols before distribution. Alternatively, some households receive their water supply from local community water cooperatives, which maintain water sources primarily for their members. The majority of these community water sources serve 100 households or fewer, and the cooperatives are actively involved in the upkeep of the areas surrounding the water sources and reservoirs. However, when researchers utilized the WHO sanitary inspection form for dug wells and springs, researchers noted that several community water sources would benefit from reevaluating their compliance with WHO standards for community deep wells and springs.

Most deep well systems are equipped with functional pumps, cover slabs, and lids to prevent contaminants from entering the well. These deep wells also feature adequate walls, aprons, barriers, and proper drainage systems. Furthermore, researchers recorded strategically positioned sanitary infrastructure within a 30-meter radius of the well, with no identified entry points to the aquifer. These findings indicate a generally positive state of deep well systems in the locality while highlighting opportunities for reinforcing adherence to WHO standards in community-dug wells and springs.

The ocular inspection of the sampled spring sources revealed that all springs exhibited adequate drainage and were free from stagnation. Many of these springs featured well-constructed protective walls or spring box structures and appropriately designed or covered air vents. Nevertheless, notable deficiencies were identified, including the absence of stormwater-diversion ditches, fencing, and barriers to prevent animal access to the springs. Additionally, researchers found the outlet pipes of most spring sources to be unclean and poorly positioned. Many of these springs lacked sanitation infrastructure within a 15-meter radius and demonstrated insufficient fencing or barriers upstream to deter the ingress of contaminants. Researchers noted visible signs of contamination within some spring boxes, and a few springs lacked sanitation infrastructure on higher ground within 30 meters, exhibiting contamination indicators such as animal presence and sediment accumulation.

Due to the considerable distance of several springs from each other, barangay residents directly connect hoses from the springs or storage tanks to their households. These hoses often showed evidence of wear, overuse, or leaking, putting them at risk of transmitting disease-causing microorganisms. Drinking water can also be dangerous until authorities properly treat and sanitation the water. Unfortunately, only one out of every three community water sources addresses the contaminants found in the reservoir, where they disinfect with chlorine before distributing it throughout the homes.

## 4 Discussion

Increased detection in microscopy compared to molecular testing can be due to false positives because microscopy is based on morphological recognition, which can be misleading by artifacts and other protozoan cysts (O'Leary et al., 2021). PCR-based molecular tests recognize *Cryptosporidium* DNA with higher specificity and species resolution but can be influenced by inhibitors in fecal and environmental samples.

The resulting prevalence of *Cryptosporidium* infection among household members in this study, with a positivity of 9.33%, emphasizes the importance of exploring plausible transmission pathways within communities. The study marks the first investigation into the prevalence of *Cryptosporidium* in a significant town in Northern Luzon, Philippines. It is worth mentioning that this recorded prevalence is lesser than that revealed in a survey carried out in the adjacent province of Ifugao, where a prevalence of 28.50% was observed among 137 individuals (Labana et al., 2018).

Researchers attribute the observed prevalence difference to the sample collection timing, which occurred during strict COVID-19 restrictions in La Trinidad. The national quarantine significantly limited school attendance and workplace presence, leading residents to stay at home and prepare food within their households primarily. This period saw the implementation of stringent public health measures, including social distancing, quarantine protocols, and extensive hygiene campaigns, all likely contributing to reducing disease transmission.

The study’s findings align with the 26% decline in foodborne infections reported by the Foodborne Diseases Active Surveillance Network (FoodNet), part of the CDC’s Emerging Infections Program, during 2020 compared to 2017–2019 (Ray, 2021). Similarly, Chen et al. (2021) demonstrated that nationwide nonpharmaceutical interventions (NPIs) implemented during the pandemic effectively reduced the prevalence of notifiable infectious diseases. A study in Ethiopia also reported increased adherence to hand hygiene protocols, significantly decreasing intestinal parasitic infections (Seid et al., 2022). These findings underscore the role of rigorous hygiene measures and movement restrictions in mitigating disease transmission.

While these public health interventions successfully reduced the spread of infectious diseases, relaxing strict protocols could lead to increased transmission. Continuous monitoring and sustained public health efforts remain essential to understanding and managing shifts in disease prevalence. Household-level factors, including individual hygiene behaviors and environmental conditions, may provide valuable insights into *Cryptosporidium* transmission dynamics.

The significant association between *Cryptosporidium* detection in human samples and companion animals suggests a potential zoonotic transmission route. This finding underscores the need for regular screening and preventive measures for companion animals to minimize household transmission risks. In contrast, the absence of a significant association between human infection and livestock presence may reflect limited direct human-livestock interactions or effective hygiene practices in households managing livestock. These results highlight the critical role of companion animals in *Cryptosporidium* transmission, particularly when infected, and reinforce the necessity of targeted interventions, including stringent hygiene practices and routine surveillance of household pets. Further research using molecular characterization of *Cryptosporidium* isolates is essential to confirm transmission pathways and identify potential zoonotic strains.

*Cryptosporidium* was significantly more common in livestock (37.27%) than in companion animals (22.44%). In Ifugao, a *Cryptosporidium* prevalence of 33.33% was reported among 12 tested dogs (Labana et al., 2018). In this study, researchers observed a prevalence of 18.22% in a larger sample of 236 dogs. While this research encompassed only a limited number of participating cats and doves, it revealed that Researchers found *Cryptosporidium* species in the fecal samples of these animals, with positivity of 14.29% in cats and 50% in other animals. Previous studies in the country have not reported this specific finding. The 14.29% positivity of *Cryptosporidium* in cats observed in this study is higher than the global average of 6% reported by Taghipour et al. (2021). This difference highlights the need for more research on local factors influencing *Cryptosporidium* prevalence in cats.

The prevalence of *Cryptosporidium* in companion animals, such as dogs and cats, can be better understood by considering the changes brought about by the COVID-19 pandemic. The pandemic altered human behaviors; due to lockdowns, social distancing measures, and remote work arrangements, people spent more time indoors and developed a greater appreciation for companion animals’ emotional support and companionship. Seventy-six percent of the households in the study have companion animals, mostly with specific breeds kept indoors. It is important to look into whether different breeds of cats and dogs show different patterns related to the spread of *Cryptosporidium*, a limitation of this study. Keeping animals indoors might help spread parasites. Understanding how people and companion animals interact can reveal the risks of spreading infections, emphasizing the need for the One Health approach. This study found a possible link between *Cryptosporidium* infection in companion animals and its occurrence in household members. The infections in the same household may be connected or share similar risk factors. However, the study did not explore the sources of infection. Although the researchers found an association, researchers did not examine the precise causes, sources, or modes of transmission of *Cryptosporidium* infection. More researchers should conduct more studies on this subject to understand these gaps.

The results indicate that several livestock species, including chicken, rabbit, pig, and cow, harbor *C. parvum* infections. Other studies conducted in the country have reported the isolation of *Cryptosporidium* infections in cows in Palawan (Laxer et al., 1988), pigs in Palawan and Ifugao (Labana et al., 2018), and chickens in Ifugao (Labana et al., 2018). To increase household income, many residents practiced backyard chicken farming. During the study, researchers found that 37.50% of the households were involved in raising chickens. Most of these chickens were allowed to roam freely, leaving droppings in different areas. Some people kept their animals in shelters made from bamboo or steel and buried their waste in the ground. These practices, particularly the lack of proper fencing for free-range chickens and the waste disposal methods for confined chickens, may have led to the high levels of *Cryptosporidium* in the chicken population. The temporary setup of these backyard farms offered limited biosecurity, making it easier for parasites to spread. Similar findings were reported by Cornell et al., 2022. The concerns about how poor animal waste disposal can lead to the spread of *Cryptosporidium* emphasize the need for greater awareness and better practices.

This study is the first to document the existence of *Cryptosporidium* infection in rabbits in the country. Rabbits are considered livestock in the Philippines, as they are a good source of healthy white meat. The rabbit industry in Benguet province is still in its early stages but shows growth potential. Researchers observed small-scale rabbit rearing during house-to-house visits. A household representative revealed in an interview that people commonly use rabbit stool samples as fertilizer in backyard gardens. Rabbit meat reared in the locality is already available on the market. Research should support and back sufficient programs and standards as the rabbit industry is a potential agricultural venture in the locality. Determining the extent of *Cryptosporidium* and other parasitic infections among this livestock is crucial for infection control and prevention to ensure safe human consumption (Zhang et al., 2012).

This study found that all 19 sampled community water source facilities from protected deep wells and protected springs tested negative for *Cryptosporidium*. From a public health perspective, this is a positive indication of less disease transmission. The *Cryptosporidium* infections observed in household members, companion animals, and livestock are most likely not attributable to contaminated water sources. This suggests that, at the time of sampling, the water sources did not contain a detectable level of *Cryptosporidium* oocysts, the infectious stage of the parasite. The present study’s limitations of a single sample collection test affected the result. Animals shed *Cryptosporidium* oocysts sporadically, and their presence in water may vary over time. Therefore, although the present findings are positive, they cannot accurately represent the long-term state of water safety. Regular monitoring and testing are essential to maintain water quality. Most administrators of the community water systems sampled articulated this need, recognizing that regular testing is the only means of determining the safety of community water sources. In this study, researchers analyzed all *Cryptosporidium* isolates through microscopy and molecular tests to assess species. Identifying *Cryptosporidium* species is crucial for gaining insight into the disease’s epidemiology (Pane and Putignani, 2022).

## 5 Conclusion and recommendations

This study provides information on the prevalence of *Cryptosporidium* species in Northern Luzon, Philippines, particularly in La Trinidad, Benguet. The findings show a lower prevalence of *Cryptosporidium* infection in household members compared to earlier regional studies, likely due to the successful public health measures during the COVID-19 pandemic. However, detecting *C. parvum* in fecal samples from companion animals and livestock emphasizes the need to monitor diseases that spread from animals to humans.

The lack of *Cryptosporidium* in water samples shows the importance of keeping water safe. However, it also suggests that other ways of spreading the disease, such as contact with infected animals or dirty surfaces, should be considered. Public health efforts should prioritize water safety, hygiene, and sanitation practices at home to minimize the risk of diseases transferring from animals to humans.

This study supports the One Health approach, which connects human, animal, and environmental health to create effective strategies for preventing and controlling diseases. Cooperation between animal and human health experts is key to understanding how diseases spread and improving public health actions. Regular testing of humans, companion animals, and livestock for *Cryptosporidium* is important to detect infections early and respond quickly. Continuing the substantial public health practices used during the COVID-19 pandemic can further reduce the spread of infectious diseases.

Public health campaigns should continue to educate people about the dangers of diseases that spread from animals to humans and the importance of practicing good hygiene. Training programs should teach proper handling and care of companion animals and livestock to minimize the risk of infection transmission. Families should also be intentional to practice their hygiene practices, including regular handwashing, proper and safe disposal of animal waste, and cleaning areas where animals reside. Authorities should educate households on preventing the transmission between animals and humans to reduce the risk of zoonotic diseases.

More research is needed to understand how *Cryptosporidium* spreads, especially the role of different environmental and behavioral factors. Studies should also look into other possible sources of infection, such as contact with animals and contaminated surfaces, beyond just water pollution. This research shows the occurrence of *Cryptosporidium* and identifies molecular species in La Trinidad, Benguet, revealing the zoonotic transmission hazards linked to *C. parvum*. This study highlights the importance of adopting a holistic One Health strategy to address and prevent infections efficiently. By combining approaches that consider human, animal, and environmental health and implementing specific public health interventions, we can effectively tackle the intricate dynamics of zoonotic transmission and reinforce public health defenses against emerging infectious diseases.

## Data Availability

The datasets presented in this study can be found in online repositories. The names of the repository/repositories and accession number(s) can be found in the article/supplementary material.
